# Metabolomics highlights biochemical perturbations occurring in the kidney and liver of mice administered a human dose of colistin

**DOI:** 10.3389/fmolb.2024.1338497

**Published:** 2024-07-10

**Authors:** I. Barla, I. V. Dagla, A. Daskalopoulou, M. Panagiotopoulou, M. Kritikaki, P. Dalezis, N. Thomaidis, A. Tsarbopoulos, D. Trafalis, E. Gikas

**Affiliations:** ^1^ Laboratory of Analytical Chemistry, Department of Chemistry, School of Science, National and Kapodistrian University of Athens, Athens, Greece; ^2^ GAIA Research Center, The Goulandris Natural History Museum, Kifissia, Greece; ^3^ Laboratory of Pharmaceutical Analysis, Department of Pharmacy, School of Health Science, National and Kapodistrian University of Athens, Athens, Greece; ^4^ Laboratory of Pharmacology, School of Medicine, National and Kapodistrian University of Athens, Athens, Greece

**Keywords:** metabolomics, colistin, drug toxicity, pathway analysis, dopamine metabolism, purine metabolism

## Abstract

**Introduction:** Colistin (CMS) is used for the curation of infections caused by multidrug-resistant bacteria. CMS is constrained by toxicity, particularly in kidney and neuronal cells. The recommended human doses are 2.5–5 mg/kg/day, and the toxicity is linked to higher doses. So far, the *in vivo* toxicity studies have used doses even 10-fold higher than human doses. It is essential to investigate the impact of metabolic response of doses, that are comparable to human doses, to identify biomarkers of latent toxicity. The innovation of the current study is the *in vivo* stimulation of CMS's impact using a range of CMS doses that have never been investigated before, i.e., 1 and 1.5 mg/kg. The 1 and 1.5 mg/kg, administered in mice, correspond to the therapeutic and toxic human doses, based on previous expertise of our team, regarding the human exposure. The study mainly focused on the biochemical impact of CMS on the metabolome, and on the alterations provoked by 50%-fold of dose increase. The main objectives were i) the comprehension of the biochemical changes resulting after CMS administration and ii) from its dose increase; and iii) the determination of dose-related metabolites that could be considered as toxicity monitoring biomarkers.

**Methods:** The *in vivo* experiment employed two doses of CMS versus a control group treated with normal saline, and samples of plasma, kidney, and liver were analysed with a UPLC-MS-based metabolomics protocol. Both univariate and multivariate statistical approaches (PCA, OPLS-DA, PLS regression, ROC) and pathway analysis were combined for the data interpretation.

**Results:** The results pointed out six dose-responding metabolites (PAA, DA4S, 2,8-DHA, etc.), dysregulation of renal dopamine, and extended perturbations in renal purine metabolism. Also, the study determined altered levels of liver suberylglycine, a metabolite linked to hepatic steatosis. One of the most intriguing findings was the detection of elevated levels of renal xanthine and uric acid, that act as AChE activators, leading to the rapid degradation of acetylcholine. This evidence provides a naïve hypothesis, for the potential association between the CMS induced nephrotoxicity and CMS induced 39 neurotoxicity, that should be further investigated.

## 1 Introduction

Colistin is typically used as a last resort antibiotic to treat infections caused by multidrug-resistant bacteria as it has a narrow therapeutic index and can cause significant toxicity, particularly to the kidneys and nervous system (Grégoire et al., 2017; Satlin et al., 2020; Mohapatra et al., 2021). It belongs to the class of antibiotics known as polymyxins, which have a unique mechanism of action that disrupts the bacterial cell membrane. This mechanism of action makes polymyxins effective against many Gram-negative bacteria that are resistant to other antibiotics, such as *Pseudomonas aeruginosa*, *Acinetobacter baumannii*, and *Klebsiella pneumoniae* (Falagas and Kasiakou, 2005). However, polymyxins are also toxic to human cells, particularly kidney cells and neuronal cells. The drug is usually given as colistimethate sodium (CMS), a prodrug of colistin (Bergen et al., 2006). The dosage regimen of CMS can vary depending on the patient’s age, weight, the severity of infection, and the method of administration (injection or inhalation). For the treatment of infections caused by Gram-negative bacteria, the usual adult dose is 3 million units daily, administered intravenously in divided doses every 8 h (Plachouras et al., 2009). An average steady-state plasma colistin concentration of 2 mg/L seems to be a reasonable target value (Nation et al., 2015). A loading dose CMS is often recommended to achieve a therapeutic concentration quickly. The loading dose can vary depending on the patient’s condition, but it is usually higher than the subsequent maintenance dose (Haseeb et al., 2021; Rychlíčková et al., 2023). The duration of treatment can also vary depending on the severity of infection and the patient’s response to therapy. According to the prescribing information for CMS injection, the maximum daily dose should not exceed 5 mg/kg of body weight (https://www.accessdata.fda.gov/drugsatfda_docs/label/2009/050108s026lbl.pdf, accessed on 26 April 2023). It is important to note that the dosage regimen for CMS should be adjusted in patients with renal impairment as the drug is primarily eliminated by the kidneys (Garonzik et al., 2011). The use of CMS should always be monitored by a healthcare professional. An approach to increase the drug efficacy is the co-administration of CMS with other antibiotics such as fosfomycin (Katip et al., 2024).

CMS, like other antibiotics, can cause side effects and toxicities, i.e., brain dysfunction and neurotoxicity. The mechanism of CMS-induced neurotoxicity is not fully understood, but it is thought to involve the drug’s ability to penetrate the blood–brain barrier and interact with neuronal cells (Inci et al., 2018; Dai et al., 2019; Ramezanzade et al., 2023). Furthermore, CMS can cause damage to the kidneys, especially if it is used for a long time or at high doses. This can lead to symptoms such as decrease in creatinine clearance, decreased urine output, proteinuria, cylinduria, swelling in the legs or feet, and shortness of breath (Gai et al., 2019; Eljaaly et al., 2021; Alotaibi et al., 2022).

*In vivo* studies of CMS nephrotoxicity have shown histological abnormalities, tubular dilation and cell necrosis, and epithelial cell vacuolation as well (Gai et al., 2019). Although CMS is primarily associated with kidney toxicity, there is some evidence to suggest that it may also have an effect on the liver (Long et al., 2022). It is important to note that the risk of toxicities may vary depending on the dose, duration of treatment, and individual patient factors. Therefore, the levels of CMS should be carefully monitored.

Despite these limitations, CMS remains a last-resort treatment against multidrug-resistant bacteria. There is limited literature on CMS toxicity; i.e., in PubMed (https://pubmed.ncbi.nlm.nih.gov/?term=colistin+induced+toxicity, last access in March 2024), the search *colistin induced toxicity* resulted in 178 published papers.

The majority of the CMS toxicity *in vivo* studies use doses even 10-folds higher than the human doses (Gai et al., 2019), to investigate the background of highly toxic conditions; therefore, our knowledge on the impact of low CMS doses is limited. In addition, metabolomics studies of colistin toxicity are limited, although *in vivo* metabolomics experiments could increase our knowledge on CMS impact, as the metabolites are molecules of universal structure and, thus, provide consistent interpretation of the evidence resulting from toxicological studies using animal-to-human translation. Until now, one metabolomics study has been conducted for the characterization of urinary metabolites as biomarkers of colistin-induced nephrotoxicity in rats (Jeong et al., 2016). Furthermore, Long et al. (2022) have studied the alteration of kidney and liver metabolome after the administration of a high CMS dose.

Based on the above, the objective of the current study is the evaluation of the biochemical impact resulting from realistic human-relevant CMS doses and from an increase of 50% that is perceived as pragmatistic . The main objectives of the study were 1) comprehension of the biochemical changes resulting from the mentioned doses; 2) determination of dose-related metabolites, as potential toxicity-monitoring biomarkers; and 3) estimation of early signs of hepatotoxicity. Therefore, an *in vivo* stimulation of CMS metabolic changes was performed by administering CMS to mice (1 and 1.5 mg/kg/day for 5 sequential days). Plasma, kidney, and liver were used for metabolomics profiling using a UPLS-MS-based platform. The doses were calculated by using the dose conversion from animals to humans (Nair and Jacob, 2016; Jacob et al., 2022). Based on the clinical experience of our team, these doses correspond to human exposure, representing the therapeutic and toxic dose.

## 2 Materials and methods

### 2.1 Animals and dosage regime

The study employed plasma, kidney, and liver samples of 15 C57Bl/6 (weight: 20–25 g; age: 8–10 weeks) mice. All *in vivo* experiments were carried out in accordance with the “Guide for the care and use of laboratory animals,” and experiments were approved by the Ethics Committee (Approval No: 574234/20-07-2020). The mice were housed and maintained according to the ARRIVE guidelines. The number of animals needed to achieve statistical power >80% was calculated using GPower 3.1. The animals were randomized into three groups (*n* = 5 for each group) as follows: 1) control (NaCl 0.9%), 2) low dose (LD) (CMS 1 mg/kg/day), and 3) high dose (HD) (CMS 1.5 mg/kg/day). The drug and the normal saline, respectively, were injected intramuscularly (i.m.) to the thigh of each laboratory animal. The administration was repeated for 5 sequential days, and on the sixth day, the laboratory animals were sacrificed.

### 2.2 Metabolite extraction from plasma and tissues

For the plasma extraction, 200 μL of the sample was mixed with 600 μL of frozen MeOH, centrifuged using a Neya 16R centrifugation apparatus (REMI, Mumbai, India) at 10,000 rpm, 5 min, 4°C, and the supernatant was stored at −80°C. For the extraction of liver and kidney samples, 100 mg of the tissue was homogenized with 1 µL of MeOH–H_2_O (1:1, v/v) solution. A Cryolys Evolution tissue homogenizer (Bertin Instruments, Rockville, MD, United States) and the homogenizing CKMix lysing kit (Bertin Corp., Rockville, MD, United States) were employed for a two-step procedure. At first, 500 μL of the solution was added in the falcon tube with the tissue, and the “hard” mode (9,600 rpm, three 20-s cycles followed by a 60-s pause) of the homogenizer was applied. Then, the blend was centrifuged, and the supernatant was obtained. For the second step, after removing the supernatant, the rest of the solution was added to the homogenizing tube with the remaining tissue and submitted to a second cycle of a “soft” mode (5,000 rpm, one 60-s cycle) homogenization. The blend was centrifuged, as before, and the supernatant was obtained and mixed with the supernatant obtained from the 1st homogenization cycle; 400 μL and 500 μL of plasma and tissue extract, respectively, were evaporated to dryness using a HyperVAC-LITE centrifugal vacuum concentrator (Hanil Scientific Inc., Gimpo, Korea), and the remaining solid was reconstituted with 150 μL of IS mix solution containing 1 ppm of yohimbine and reserpine in MeOH–H_2_O (1:1, v/v). Yohimbine and reserpine are pharmaceutical compounds that are not normally detected in organisms and were used for multi-IS-based signal correction.

### 2.3 UPLC-MS analysis

The samples were analyzed using a Dionex UltiMate 3000 RSLC (Thermo Fisher Scientific, Dreieich, Germany) UPLC system, coupled to a maXis Impact QTOF mass spectrometer (Bruker Daltonics, Bremen, Germany) equipped with an electrospray ionization source. The chromatographic column used was Acclaim RSL C18 column (2.1 × 100 mm, 2.2 μm, Thermo Fisher Scientific), and elution of the analytes was performed with gradient conditions, by a ramp increase of the organic mobile phase. The DIA methodology (bbCID mode, in Bruker terminology) was selected for MS acquisition. A detailed description of the UPLC conditions and the parameters of ESI and MS are included in the Supplementary Material, § Experimental Condition.

### 2.4 Data processing

The MZmine 4.9 software was selected for untargeted peak-picking (Pluskal et al., 2010). The QCRLSC (Luan et al., 2018) algorithm (via the StatTarget platform) (Luan et al., 2018) and the NOMIS algorithm [via NOREVA 2.0 platform (Fu et al., 2022)] were employed for QC- and IS-based signal correction, respectively. RamClustR was used for the assignment of pseudo-MSMS clusters (Broeckling et al., 2014), and MCID (http://www.mycompoundid.org/mycompoundid_IsoMS/) (Huan et al., 2015) and HMDB (Wishart et al., 2018) online libraries were used for metabolite annotation. SIMCA 14.1 (Umetrics, Upsala, Sweden) and MetaboAnalyst 5.0 (https://www.metaboanalyst.ca) (Pang et al., 2021) platforms were used for statistical analysis. Moreover, TASQ Client 2.1 software (Bruker Daltonics, Bremen, Germany) was used for the screening of the raw MS data, for the determination of metabolites involved in purine metabolism. Further description of the pre-processing workflow is included in the Supplementary Material.

### 2.5 Statistical analysis design

The final feature tables were submitted to the following statistical tests. Initially, the groups were submitted to pairwise comparisons, i.e., C vs. LD and LD vs. HD, using PCA and OPLS-DA. Pairwise comparisons were selected as they provided several advantages:

• The comparison of C and LD provided an insight into the biochemical impact of CMS on the metabolome, and so far, to the best of our knowledge, this issue has not been addressed by the existing literature.
• The comparison of LD and HD provided an image of what happens when the CMS dose is 50% increased. It is worth mentioning that low-dose increases display a realistic scenario of clinical practice, and considering that CMS is nephrotoxic, increasing the circulating levels of CMS could result in latent or severe toxicity outcomes.
• The pairwise comparisons provide the ability to use ortho-based methodologies, i.e., OPLS-DA, and thus remove the impact of confounders on the metabolomics profiles, acquiring more interpretable results.
• Pairwise comparisons can be combined with methodologies such as SUS plot. The SUS plot compares two ortho-models simultaneously and provides information about their common and different trends, which is discussed in detail below.

Considering that the CMS nephrotoxicity is dose-related, based on the existing literature, the metabolites differentiated in LD and HD could reveal latent signs of toxicity and even facilitate toxicity monitoring. For further investigation, OPLS-DA was used to develop the SUS plot that allowed structure comparison of the two states.

PLS regression models were used to investigate if there is a linear correlation between the dose and the metabolomics alterations, as has been observed by Nguyen et al. (Gai et al., 2019) when administrating higher doses of CMS. Acknowledging that the linear correlations provide limited explanation of the dose–response phenomena, the study focused on the linearly dose-related metabolites as their alterations were more easily interpretable, and, most importantly, they could lead to monitoring biomarkers, i.e., metabolites whose their levels are altered by the dose increase in a consistent way and could betray the stage or the development of toxicity.

Finally, univariate Receiver Operating Characteristic (ROC) curve analysis was used for pairwise comparisons to validate the prediction ability of the variables that were statistically significant in the multivariate tests.

## 3 Results

### 3.1 Statistical analysis results

**FIGURE 1:**
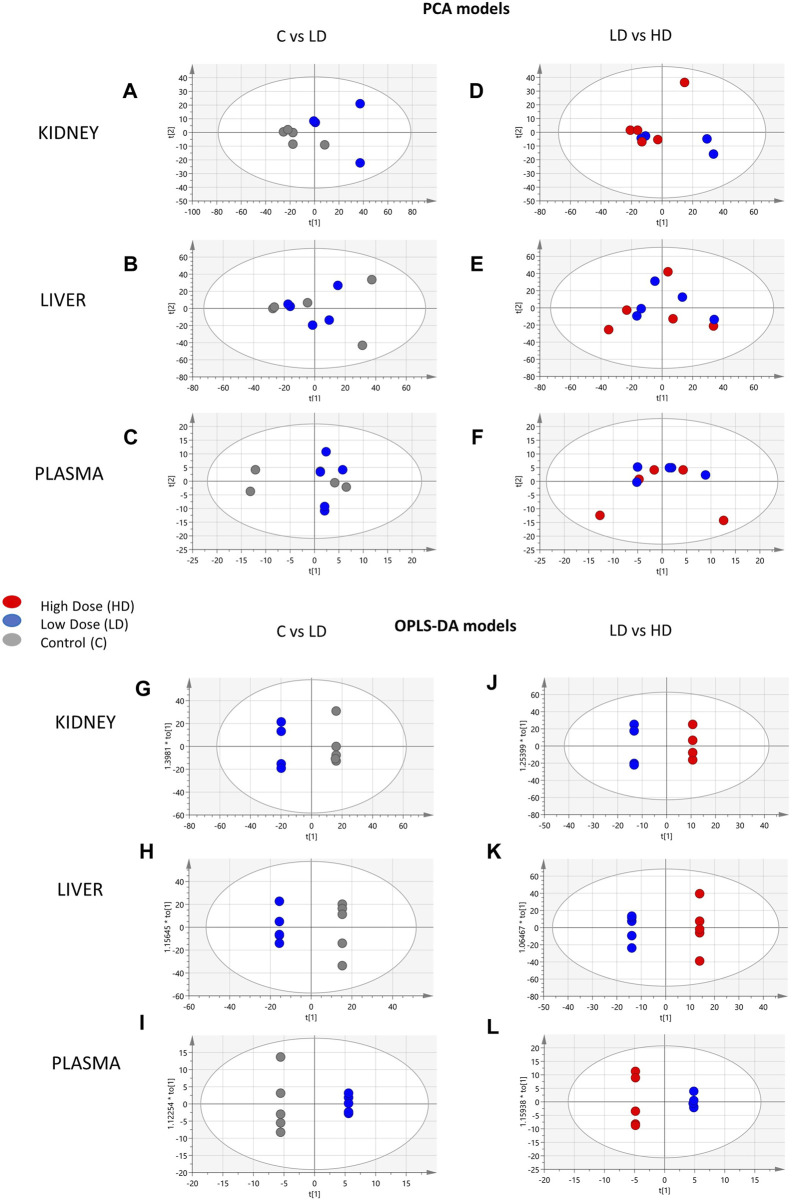
Summary of the PCA and OPLS-DA results of the ESI^+^ datasets. PCA scores’ plots of the following: **(A)** kidney, C (*n* = 5)-LD (*n* = 5); **(B)** liver, C (*n* = 5)-LD (*n* = 5); **(C)** plasma, C (*n* = 5)-LD (*n* = 5); **(D)** kidney, LD (*n* = 5)-HD (*n* = 5); **(E)** liver, LD (*n* = 5)-HD (*n* = 5); **(F)** plasma, LD (*n* = 5)-HD (*n* = 5). OPLS-DA scores’ plots: **(G)** kidney, C (*n* = 5)-LD (*n* = 4); **(H)** liver, C (*n* = 5)-LD (*n* = 5); **(I)** plasma, C (*n* = 5)-LD (*n* = 5); **(J)** kidney, LD (*n* = 5)-HD (*n* = 5); **(K)** liver, LD (*n* = 5)-HD (*n* = 5); **(L)** plasma, LD (*n* = 5)-HD (*n* = 5).

The control (C), the low dose (LD)-treated, and the high dose (HD)-treated mice were compared in pairs to detect classification trends correlated 1) with the drug administration (C vs. LD) and 2) with the drug dose (LD vs. HD). Initially, PCA exhibited clear separation of C and LD mice in the kidney (Figure 1A) but there was no separation observed either in C–LD comparison, or in the plasma and liver (Figures 1B–F). The confidence and performance of the developed models were estimated through misclassification and permutation testing and model ROC analysis, and the results, along with the figures of merit, are presented in Supplementary Table S1.

The main objective of the study was to investigate the alterations occurring by dose increase, and thus, the study was focused on the differentially expressed variables acquired from the LD–HD comparisons. Those with Variable Importance in the Projection (VIP) > 1.2 were employed for univariate ROC curve analysis to detect the variables that responded to the dose increase. The number of variables with Area Under Curve (AUC) value >0.8 were as follows: 251 variables for kidneys (49 upregulated in the HD), 345 variables for the liver (136 upregulated in the HD), and 47 variables for plasma (28 upregulated in the HD).

**FIGURE 2:**
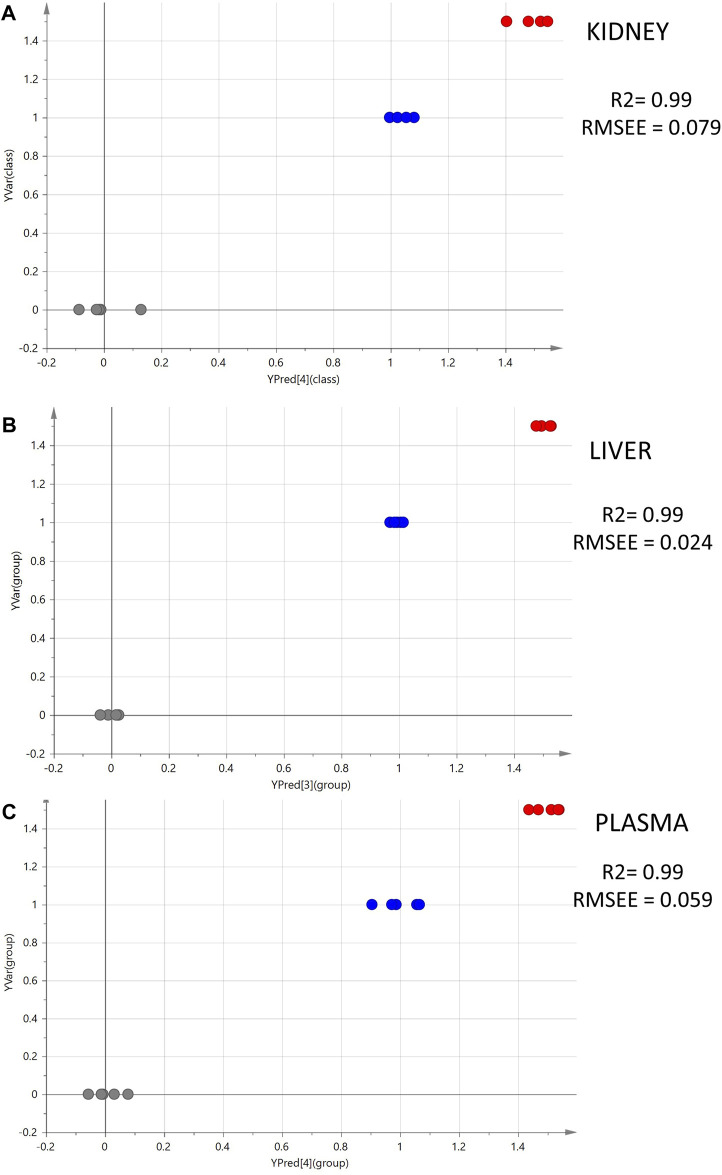
Summary of PLS analysis. Observed vs. predicted plots of ESI^+^ datasets: **(A)** kidney; **(B)** liver; **(C)** plasma. The gray spots represent the C samples (CMS 0 mg/kg, *n* = 5); the blue spots represent the LD samples (CMS 1.0 mg/kg, *n* = 5); and the red spots represent the HD samples (CMS 1.5 mg/kg, *n* = 5).

Moreover, PLS models were employed to investigate the linear correlation between the three levels of administered CMS (0, 1, and 1.5 mg/kg) and the alteration occurring to the metabolomics profiles of the mice. The excellent linearity (*R* ^2^ > 0.99) and the low root means square error of estimation of the PLS models (Figure 2) prove that there is a linear correlation between the administered dose and the resulting metabolomics alterations.

**FIGURE 3:**
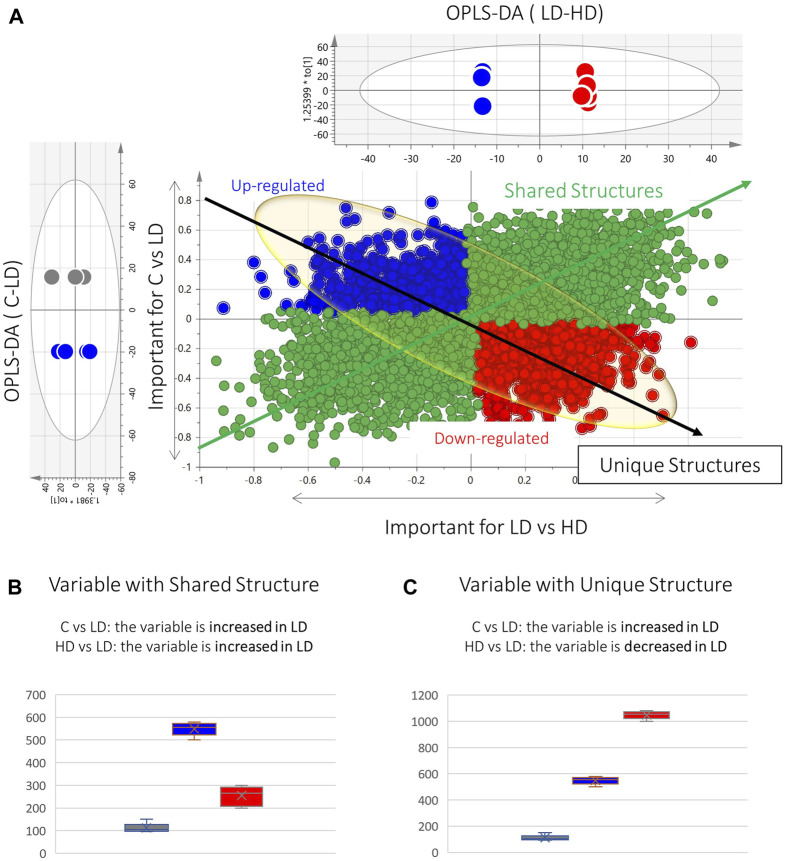
Graphical description of the SUS plot basic information. **(A)** SUS plot developed by the OPLS-DA models of C–LD and LD–HD of the kidney ESI^+^ dataset. The green spotted area represents the variables of the shared structure. The blue and red spotted areas represent the variables of the unique structure. At the edges of the black arrow of unique structures exist the statistically significant dose–response variables (red: upregulated, blue: downregulated). **(B)** Box-plot representing a variable of the shared structure, which shows increased levels in LD (blue) but decreased levels in C (gray) and HD (red). **(C)** Box-plot representing an example of an upregulated unique structure variable, which is increased in LD (blue) compared with C (gray) and in parallel is decreased in LD, compared with HD (red).

To elaborate on this observation, the OPLS-DA models of C–LD and LD–HD comparisons were used to build shared and unique structure plots, known as SUS plots, aiming to highlight the most highly dose-correlated variables. The SUS plot describes the correlation of the predictive variables afforded by the two OPLS-DA models, by plotting the loadings of both against each other. In this case, the two OPLS-DA models share a common group, the LD group. The SUS plot in Figure 3A represents the loadings of C–LD and LD–HD for the dataset of kidney, ESI^+^. The features at the edges of the y-axis are discriminant for the model C–LD, and those at the edges of the x-axis are discriminant for the model LD–HD. The features existing in the diagonal orientation from the lower left to the higher right (Figure 3A, green arrow) are those that present shared structures, whereas those existing in the diagonal orientation from the higher left to the lower right (Figure 3A, black arrow) present unique structures. As the LD is the common group in the two OPLS-DAs, the variables of the shared structure show the same regulation in the LD group, in both C–LD and LD–HD comparisons. For example, the variable in Figure 3B shows increased levels in LD samples and decreased levels in C and HD samples; so, as it is always increased in the LD group, it is considered a variable of shared structure. Conversely, the variable in Figure 3C shows increased levels in the LD group, compared with the C-group levels, and in parallel shows decreased levels in the LD group when compared with the HD group. Thus, it is considered to be a variable of unique structure. The features of the unique structure seem to express dose–response, whereas those of the shared structure do not provide meaningful information.

Aiming to determine variables showing linear correlation to the dose, a pipeline of three steps was followed: 1) at first, the variables existing at the edges of the shared-structure-diagonal (of the SUS plot) were selected; 2) then, they were submitted to pairwise (C–LD and LD–HD) univariate ROC curve analysis, and those having AUC > 0.8 in both comparisons were retained; and 3) they were used to develop regression curves, as shown in Supplementary Figures S1, S2. This procedure resulted in 16 dose–response variables, with *R* ^2^ > 0.7; nine of them were upregulated in correlation to the dose (Table 1).

**TABLE 1:** Summary of information for the dose–response variables resulting from the SUS plot procedure.

Dataset	M/Z	RT	R2	VIP (HD–LD)	AUC (HD–LD)	*p*-value (HD-LD)	Regulation (HD)	VIP (C–LD)	AUC (C–LD)	*p*-value (C–LD)	Regulation (LD)
KIDNEY(−)	205.994	0.8	0.78	1.2	0.85	0.08	↑	1.1	1	0.02	↑
KIDNEY(+)	125.0185	3.6	0.79	1.2	1	0.02	↑	1.1	0.85	0.12	↑
KIDNEY(+)	175.0059	1.5	0.84	1.3	0.8	0.19	↓	1.0	0.9	0.03	↓
KIDNEY(+)	224.5468	6.9	0.79	1.4	0.95	0.03	↑	1.0	0.9	0.08	↑
KIDNEY(+)	262.0619	4.4	0.82	1.3	0.9	0.09	↑	1.1	1	0.03	↑
KIDNEY(+)	278.0401	3.6	0.77	1.3	0.85	0.24	↑	1.2	0.95	0.02	↑
LIVER(−)	239.0422	14.7	0.73	1.3	0.8	0.1	↓	1.2	0.8	0.16	↓
LIVER(+)	126.0923	5.2	0.71	1.7	1	0.01	↑	1.2	0.76	0.22	↑
LIVER(+)	195.1244	15.1	0.76	1.5	0.88	0.03	↑	0.9	0.8	0.48	↑
LIVER(+)	206.1408	8.9	0.75	1.6	0.88	0.10	↑	1.1	0.72	0.19	↑
LIVER(+)	222.1616	10.2	0.78	1.8	0.84	0.10	↑	1.2	0.76	0.18	↑
LIVER(+)	241.1771	15.4	0.87	1.5	0.88	0.12	↓	1.1	0.72	0.28	↓
LIVER(+)	254.0957	12.7	0.75	1.4	0.84	0.19	↓	1.1	0.72	0.26	↓
LIVER(+)	348.3154	7.5	0.84	1.3	0.88	0.09	↓	1.6	0.96	0.03	↓
LIVER(+)	359.3187	16.1	0.72	2.2	1	0.00	↓	1.4	0.80	0.12	↓
LIVER(+)	399.2547	7.4	0.76	1.6	0.84	0.05	↓	1.7	0.92	0.02	↓

The (R2) column includes the R2 factor of linearity resulting from the developed regression curves.

### 3.2 Variable selection and identification

**FIGURE 4:**
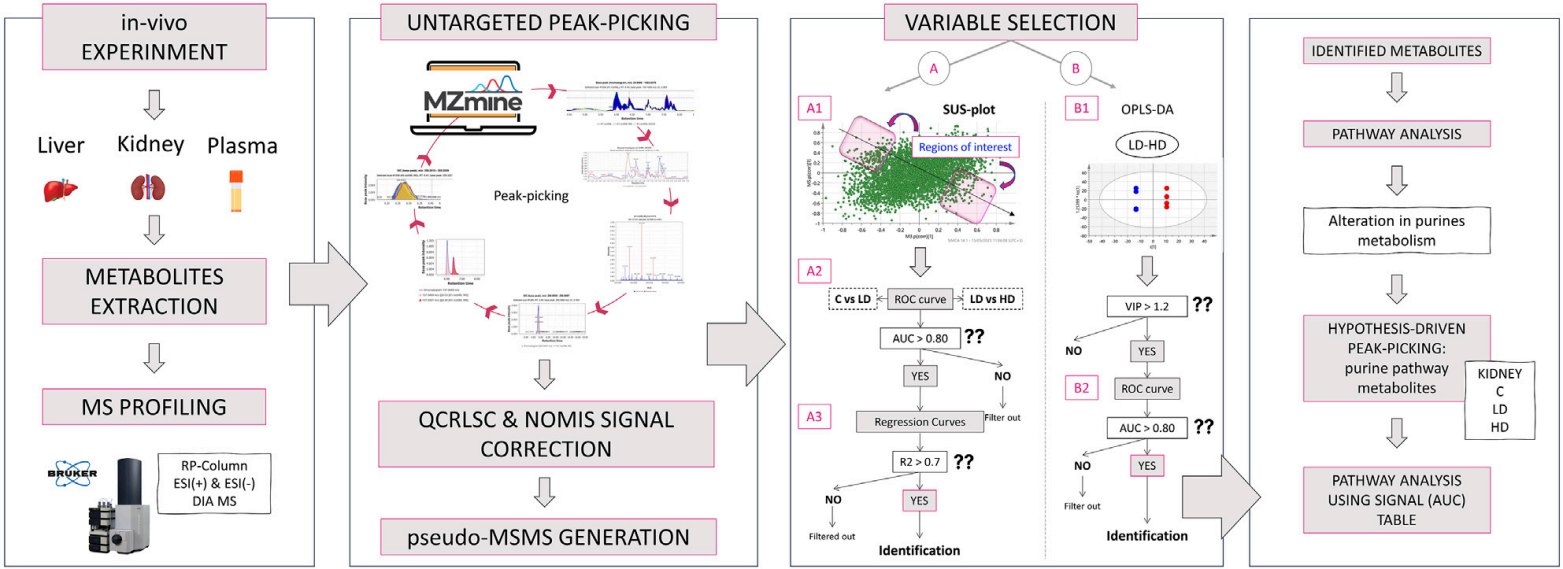
Graphical description of the applied metabolomics workflow: Initially, the *in vivo* experiment simulated the impact of 1-mg/kg and 1.5-mg/kg CMS in mice. The liver, kidney, and plasma samples were used for metabolite extraction, and the analysis was conducted with a Bruker maXis Impact QTOF MS, using RPLC and ESI^+/−^ ionization. The MS was acquired with the DIA methodology. Then, the raw data were used for untargeted peak-picking, and the feature list was submitted to QC- and IS-based signal correction. RAMclustR was used for the generation of pseudo-MSMS. Then, the features were used for variable selection performed with two ways: i) extraction of dose–response variables using the SUS plot procedure and ii) extraction of dose-correlated variables combining multivariate (OPLS-DA) and univariate (ROC) models of the LD–HD comparison. The most discriminant variables were subjected to identification. The identified metabolites were used for a naïve pathway analysis. The results showed an alteration in renal purine metabolism, and thus, a targeted peak-picking of specific metabolites was applied to the raw data on kidneys. The results were used for a new semi-quantitative pathway analysis.

The identification was statistically driven by the results of the pairwise comparison of LD–HD and also by the results of the SUS plot procedure. The pipeline followed for the current metabolomics study is described in Figure 4. Thirty-four statistically important features were attributed to known metabolites, as shown in Table 2. The identified metabolites were submitted to pathway analysis, showing extended alteration of purine metabolism in the kidneys. Thus, an additional hypothesis-driven peak-picking, focusing on the metabolites involved in the purine metabolism pathway, was performed. The screening list of the investigated metabolites is provided in Supplementary Table S3. For this step, the C, LD, and HD groups of kidney samples were used, and 27 metabolites were finally identified.

**TABLE 2:** Summary of the identified metabolites.

mz_rt	Precursor	Common name	Mass error (Da)	Initial score	Fit score	Dataset	1reaction	AUC (LD–HD)	Regulation^a^
*192.0526_4.06*	[M−H]−	Glycolic acid	<0.001	1	0.675	KIDNEY	[+(C_5_H_5_N_5_ − H_2_O)]	1	↓
*211.9876_3.93*	[M−H]−	L-Aspartyl-4-phosphate	−0.009	1	0.383	KIDNEY	-	1	↓
*125.0185_3.57*	[M+H]+	(R)-N-Methylsalsolinol	−0.002	1	0.931	KIDNEY	[+CO2]	0.85	↑ Dose-dependent
*136.0652_1.76*	[M+H]+	Adenine	0.003	1	0.458	KIDNEY	-	1	↓
*159.0317_1.5*	[M+Na]+	Hypoxanthine	0.004	1	0.494	KIDNEY	-	1	↑
*175.006_1.51*	[M+K]+	Phenylacetic acid	−0.010	1	0.302	KIDNEY	-	0.9	↓ Dose-dependent
*194.0826_5.37*	[M+H]+	2-Methylhippuric acid	0.001	1	0.707	KIDNEY	-	1	↓
*232.0382_5.38*	[M+K]+	2-Methylhippuric acid	0.001	1	0.627	KIDNEY	-	1	↓
*256.122_5.57*	[M+Na]+	Hydroxypropionylcarnitine	0.006	1	0.321	KIDNEY	-	1	↓
*262.062_4.45*	[M+H]+	2,8-Dihydroxyadenine	−0.006	1	0.508	KIDNEY	[+C_4_H_2_N_2_O]	1	↑ Dose-dependent
*269.0943_2.25*	[M+H]+	Inosine	0.006	0.998	0.822	KIDNEY	-	1	↓
*272.0946_5.57*	[M+H]+	Deoxycytidine	0.007	1	0.858	KIDNEY	[+CO_2_]	1	↓
*273.0901_1.5*	[M+Na]+	5-Methoxytryptophan	0.001	1	0.758	KIDNEY	[+O]	0.95	↓
*278.0404_3.57*	[M+H]+	Dopamine-4-sulfate	0.007	1	0.641	KIDNEY	[+CO_2_]	0.95	↑ Dose-dependent
*307.0513_2.24*	[M+Na]+	D-Glucurono-6,3-lactone	−0.002	1	0.698	KIDNEY	[+C_5_H_4_N_2_O]	0.95	↑
*348.0774_1.6*	[M+H]+	2′-Deoxyguanosine 5′-monophosphate	0.007	1	0.713	KIDNEY	-	1	↓
*371.1139_1.84*	[M+K]+	4-Hydroxynonenal	0.004	0.977	0.666	KIDNEY	[+C_6_H_8_O_6_]	1	↓
*520.3444_8.18*	[M+H]+	LysoPC(18:2(9Z,12Z))	0.005	1	0.519	KIDNEY	-	1	↑
*113.0361_1.09*	[M+H]+	Dihydrouracil	0.002	1	0.122	LIVER	[−CH_2_]	0.92	↓
*145.0505_1.91*	[M+H]+	3-Methylglutaconic acid	0.001	1	0.777	LIVER		0.92	↑
*149.1182_5.93*	[M+H]+	3-Hydroxyisoheptanoic acid	0.001	1	0.221	LIVER	[+H_2_]	0.92	↓
*188.0695_2.69*	[M+H]+	Indoleacrylic acid	−0.001	1	0.725	LIVER	-	0.92	↑
*241.1771_15.35*	[M+K]+	Spermine	−0.002			LIVER	-	0.72	↓ dose-dependent
*254.0957_12.56*	[M+Na]+	Suberylglycine	−0.004	1	0.689	LIVER	-	0.72	↓ dose-dependent
*256.0920_3.83*	[M+K]+	Propionylcarnitine	−0.003	1	0.59	PLASMA	-	0.85	↑
*278.0352_3.57*	[M+H]+	L-DOPA sulfate	0.002	1	0.65	PLASMA	-	0.92	↑
*254.0949_5.04*	[M+H]+	Neopterin	0.006	1	0.75	PLASMA	-	0.88	↑
*229.1555_1.65*	[M+H]+	L-isoleucyl-L-proline	0.000	1	0.79	PLASMA	-	1	↑
*222.0898_6.78*	[M−H]−	5-Methyldeoxycytidine	1.000	1	0.69	PLASMA	[−H_2_O]	0.98	↑
*239.1498_4.34*	[M+H]+	Homoanserine	−0.001	1	0.7	PLASMA	[−O]	1	↓
*515.1538_6.72*	[M−H]−	S-Adenosylhomocysteine	0.002	1	0.74	PLASMA	[+C_5_H_8_O_4_]	1	↑
*264.0569_3.23*	[M+H]	N-acetyl-S-(3-oxo-3-carboxynpropyl) cysteine	0.006	1	1	LIVER	[−H_2_]	1	↑
*154.0705*	[M+H]+	Dopamine	0.002	1	0.77	LIVER	-	0.79	↑
*206.1405_10.45*	[M+NH4]+	Nonic acid	0.001	1	0.76	LIVER	-	1	↓

The (1reaction) column provides information for those metabolites that were identified as products of metabolite metabolism.

^a^ The arrows (**↑**, ↓) refer to the levels of the metabolites in the HD group when it is compared with the LD group. ↑/↓ Dose-dependent refers to the metabolites that are dose-related and detected up (↑)/down (↓) regulated by the increase in the dose.

### 3.3 Pathway analysis

**FIGURE 5:**
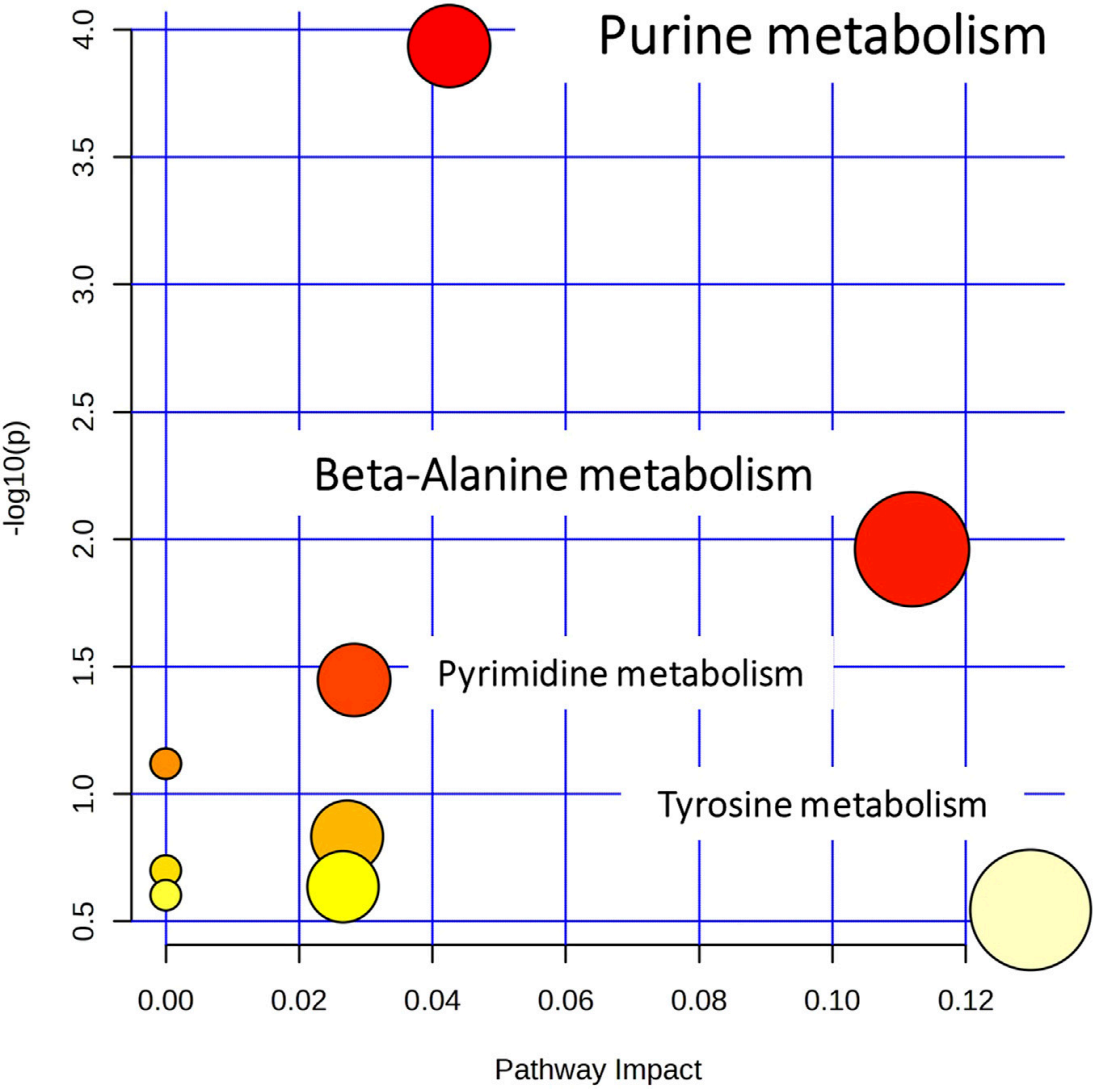
Overview of pathway analysis, showing all matched pathways according to the *p*-values from the pathway enrichment analysis and pathway impact values from the pathway topology analysis.

The pathway analysis was performed in two steps, using the *Pathway Analysis* module of MetaboAnalyst 5.0. Initially, all the identified metabolites, despite the biosample that they were detected in, were submitted to a naïve pathway analysis. This step pointed out extended dysregulation in purine metabolism, as described in Figure 5. Therefore, the metabolites participating in purine metabolism were targeted and determined in the raw MS data of all samples (kidney, plasma, and liver) and all groups (C, LD, and HD). The list of metabolites that were searched is available in Supplementary Table S3.

A higher number (27) of purine metabolism metabolites were detected in the kidney dataset, and therefore, the pathway analysis focused on kidneys. The peak area signals of the detected metabolites were used instead of concentration in the corresponding module of the Pathway Analysis tool. As this module is capable of pairwise comparisons, C–LD and LD–HD were investigated separately. In the C–LD comparison, 17/65 metabolites were altered, providing a *p*-value (FDR) = 0.04, in contrast to the LD–HD comparison that showed 17/65 altered metabolites as well, but a *p*-value (FDR) = 0.37, indicating that purine metabolism was highly influenced by the LD, but there was no further impact by the dose increase.

## 4 Discussion

This study was designed to enrich the existing knowledge of the metabolic alterations related to CMS. So far, most of the *in vivo* studies have used significantly high CMS doses to invoke the drug’s toxicity. For instance, the most recent experiment by Nguyen et al. induced *in vivo* CMS toxicity by administering 25 mg/kg and 50 mg/kg (Long et al., 2022) at low and high doses, respectively, which are 10-fold higher than the recommended human dose (2.5–5 mg/kg/day). The high-dosing approaches provide ample information on biochemical alterations occurring in extremely toxic conditions, offering clear evidence of the triggered pathways leading to the observed clinical symptoms. Conversely, such dosing regimens are never met in the clinical setting, where the administered drug levels are strictly regulated and immediately taken care of either by lowering or even by stopping the administration. However, CMS remains the last-resort antibiotic for patients infected by multidrug-resistant bacteria; thus, metabolic dysregulations caused even by the normal dosing schemes should be investigated.

Anticipating the early and frequently latent biochemical alterations provoked by the drug will provide the ability to adjust the curation protocol by reducing/stopping the administration. Under this notion, the current study attempted to simulate *in vivo* the metabolic alterations caused by the CMS doses that are comparable with those administered to humans. Thus, 15 mice were separated into three equal groups and received 0 mg/kg (C—the control group), 1 mg/kg (LD), and 1.5 mg/kg (HD) of CMS. The doses administered to mice were calculated based on the dose conversion between humans and animals. The study examined plasma, kidneys, and livers to investigate the latent background of colistin nephrotoxicity and the impact of the drug on the circulatory system and on the liver as well.

An Reversed Phase Liquid Chromatography—High Resolution Mass Spectrometry (RPLC-HRMS)-based metabolomics protocol was employed to analyze the samples, whereas an array of univariate and multivariate methodologies was combined for the statistical process. The PCA and OPLS-DA focused on the pairwise comparisons of the C–LD and LD–HD groups. PCA showed that the most significant separation trend was observed in the kidneys of those in the C–LD group. This implies that the drug triggers important metabolic alterations in kidneys, even at the lower doses, when there are no clinical data that could indicate toxicity. The OPLS-DA models classified efficiently the C–LD and LD–HD groups, providing satisfactory figures of merit.

Interestingly, the liver dataset offered the highest number of important features in both OPLS-DA and ROC curve analysis, and most of them were downregulated with the increase of the drug. However, only nine of the liver’s differentiated variables were finally identified, speculating that the drug impairs the liver metabolism, dysregulating metabolite derivatives that remain unknown. In addition, the PLS models proved the existence of linear correlations between the dose level and the alterations expressed in mice metabolomic profiles. The PLS models were only used to verify the linear correlation of metabolomics profile and CMS dose. The PLS results were not exploited for the variable selection, as PLS is a more complex model that encompasses three groups, whereas the pairwise OPLS-DA model affords more interpretable results.

**FIGURE 6:**
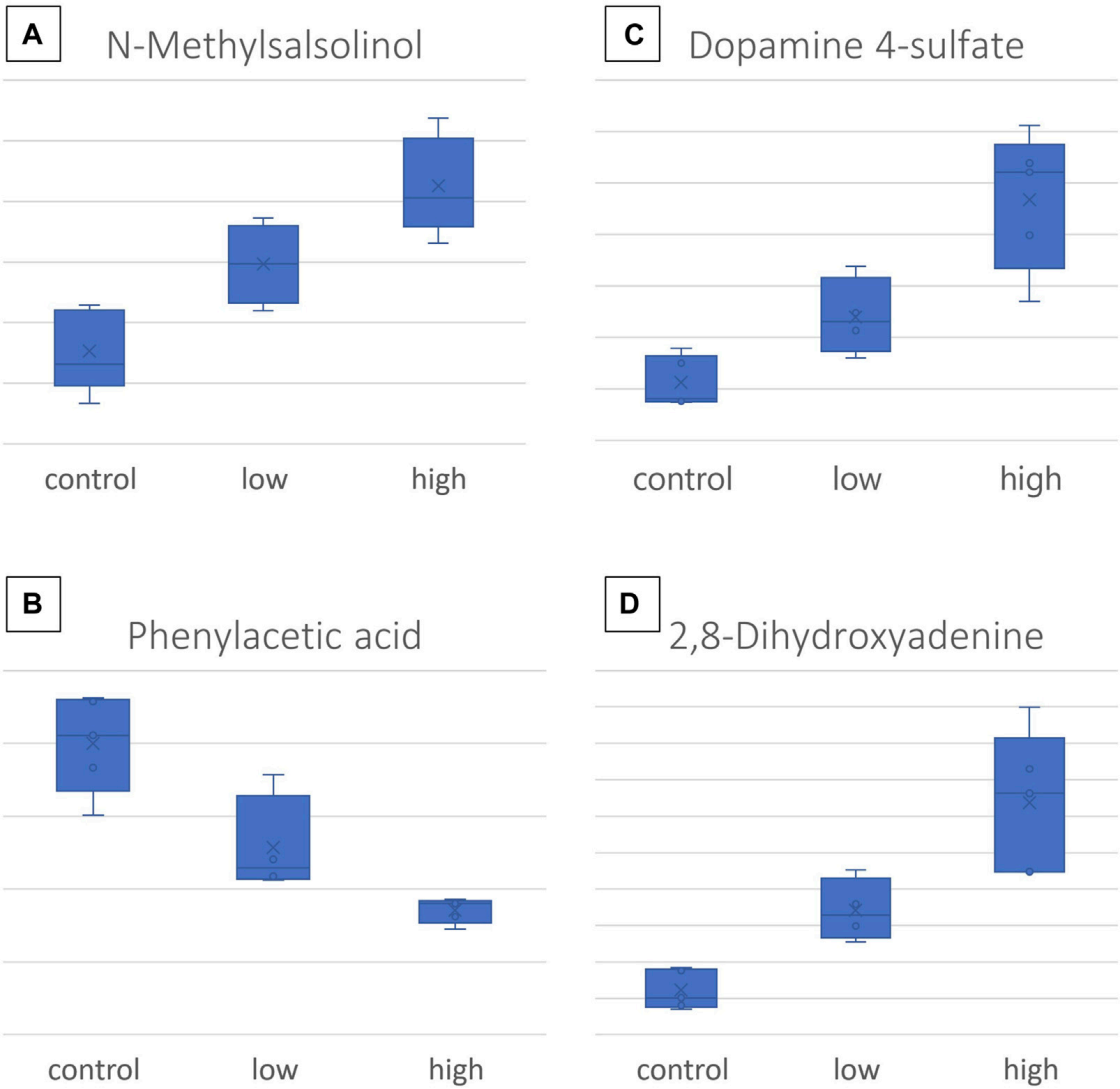
Box-plots of the most important CMS dose-responding metabolites: **(A)** N-methylsalsolinol, detected in the kidney; **(B)** dopamine-4-sulfate, detected in the kidney; **(C)** phenylacetic acid, detected in the kidney; **(D)** 2,8- dihydroxyadenine, detected in the kidney.

Concomitantly, the SUS plot was used to investigate the existence of metabolites that are linearly correlated to the dose and highlighted 16 dose-related variables, with an ROC curve AUC of >0.8 for both C–LD and LD–HD comparisons of kidneys and livers (Supplementary Figures S2, S3). In the plasma case, none of the variables resulting from the SUS plot passed the ROC curve analysis threshold, and therefore, the variables were not used to generate regression curves. Six of the dose-correlated variables were identified: suberylglycine (liver, ↓, *R* ^2^ = 0.75), spermine (liver, ↓, *R* ^2^ = 0.87), (R)-N-methylsalsolinol (kidney, ↑, *R* ^2^ = 0.79), phenylacetic acid (kidney, ↓, *R* ^2^ = 0.85), 2,8-dihydroxyadenine (kidney, ↑, *R* ^2^ = 0.82), dopamine 4-sulfate (kidney, ↑, *R* ^2^ = 0.78), and examples of their box-plots are shown in Figure 6.

Regarding the LD–HD comparison, 14, 9, and 7 differentially expressed metabolites were identified in the kidney, liver, and plasma, respectively, as summarized in Table 2. In the kidney, 78% of the metabolites were decreased in the HD group, whereas in plasma, 71% were increased. The metabolites that exhibited a higher relevance to the current case are further discussed below.

### 4.1 Alterations in dopamine regulation

The administration of CMS provoked changes in four metabolites that belong to the dopamine biochemical pathways, i.e., dopamine (DA, liver, ↑), dopamine-4-sulfate (DA-4-S, kidney, dose-increased), L-DOPA sulfate (L-DOPA-S, plasma ↑), and N-methyl-R-salsolinol (M*N*RSal, kidney, dose-increased). Differentiation of these metabolites provides a latent sign of CMS-induced dysregulation of dopamine mechanisms, that is, generalized, as the alterations were observed in the plasma, kidney, and liver as well.

The M*N*RSal, which showed elevated response to the drug dose, is an endogenous neurotoxin, related to cell apoptosis. M*N*RSal is the enzymatic product of R-salsolinol (R-Sal), which in turn is formed by DA under the action of R-Sal synthases. M*N*RSal has been detected in the urine of patients with Parkinson’s disease and is considered more toxic than R-Sal (Cao et al., 2021). M*N*RSal presents apoptotic action, and it is suggested that the toxin impairs the mitochondrial permeability transition by reducing the mitochondrial membrane potential, resulting in increased release of apoptotic factors, such as cytochrome *c*, into the cytoplasm. Moreover, the toxin activates the caspase-3, which also induces cell death (Akao et al., 2002). Furthermore, M*N*RSal degradation products inhibit the mitochondrial complex-I causing apoptosis and increase the ROS as well (Cao et al., 2021). A realistic hypothesis is that the increasing trend of the toxin in the kidney, following the increase of CMS, indicates early apoptosis in renal cells that could lead to severe kidney injury. This hypothesis is amplified by the results of a recent study that estimated the dose-responding nephrotoxicity of CMS on human embryonic kidney cells. The mentioned study showed dose-dependent renal cytotoxicity, determined by renal cell viability (Mektrirat et al., 2023).

DA-4-S showed increased levels, in response to the drug dose in the kidney, whereas L-DOPA-S was increased in plasma as well. The sulfonation locus of the endogenous/exogenous phenols and catechols, i.e., DA and L-DOPA, probably happens in the upper gastrointestinal track, where the responsible enzymes are mainly expressed (Itäaho et al., 2007). It should be noted that the sulfated forms of DA are predominant in human blood and represent 90% of the total DA (Itäaho et al., 2007). Furthermore, the sulfonation of DA is pivotal for metabolites binding with its receptors (Itäaho et al., 2007). The increased levels of DA-4-S and L-DOPA-S in plasma and kidneys suggest elevated biosynthesis of DA. DA is a natriuretic hormone and regulates sodium levels, inducing sodium excretion and constraining its reabsorption at the proximal tubule (Choi et al., 2015). Thus, excessive action of DA leads to limited levels of circulatory sodium, a condition that is linked with the occurrence of hypotension (Alshahrani et al., 2017). Additionally, it has been speculated that impairment of the estimated glomerular filtration rate (eGFR) is associated with neurological adverse effects, i.e., the limited eGFR leads to increased circulation levels of uremic toxins and kidney hormones that end up in the dopaminergic system of the brain (De Donato et al., 2022).

DA was also found elevated in the liver of the HD group. It is worth mentioning that elevated levels of DA are associated with the liver fatty acid (FA) metabolism occurring in the mitochondria of hepatocytes. The regulation of FA metabolism depends on the expression of carnitine palmitoyl transferase (CPT) I and CPT II. The catecholamines as DA induce the CPT gene expression in the hepatocytes, inducing ketogenesis. Thus, the increase of DA in CMS mice indicates dysregulation in liver FA metabolism (Jensen et al., 2013). The increased ketogenesis is also linked to hypoglycemia. The acute increase in ketones leads to nausea, vomiting, pain, lethargy, and even coma, whereas chronic ketosis can cause hepatic transaminase elevation (Drachmann et al., 2021).

### 4.2 Downregulation of renal phenylacetic acid

Phenylacetic acid (PAA) exhibited opposite response to the drug dose, in the renal tissue, i.e., metabolite decreasing levels in response to CMS increase. PAA is produced by phenylalanine degradation and is considered to be a uremic toxin. Based on existing literature, the circulatory levels of PAA have been increased in patients with chronic kidney disease (CKD) (Scholze et al., 2007); however, in the current case, no alteration of PAA plasma levels was detected. In addition, the metabolite inhibits the expression of the inducible nitric oxide synthase (iNOS) in mononuclear leukocytes in patients with end-stage renal failure (Jankowski et al., 2003). iNOS is expressed when the cells are triggered by proinflammatory cytokines and produce nitric oxide (NO) as a critical response of the immune system. In the current case, PAA was found to be decreased by CSM, speculating failure to balance the expression of iNOS leading to elevated levels of NO in the kidney. The overexpression of iNOS is linked to a variety of pathological complications such as septic shock and pain (Cinelli et al., 2020). Furthermore, the decreased renal PAA levels suggest kidney impairment resulting in the limitation of renal filtration ability. Thus, the circulatory substances do not pass from blood to the kidneys but accumulate in the circulation.

### 4.3 Upregulation of renal 2,8-dihydroxyadenine

The accumulation of 2,8-dihydroxyadenine (2,8-DHA) renal levels by CMS dosing is worth mentioning finding. 2,8-DHA is an adenine metabolite, accumulated in cases of adenine phosphoribosyl-transferase deficiency, which is a rare autosomal metabolic disorder, associated with uric acid’s metabolism. 2,8-DHA exhibits low solubility; thus, its overexpression leads to the formation and precipitation of urinary crystals and kidney stones, leading to urolithiasis or nephropathy (George et al., 2017).

### 4.4 Downregulation of liver suberylglycine

Suberylglycine decreased by CMS administration in the liver tissue. There is limited literature concerning this substance. The metabolite is normally occurring as a product of fatty acid metabolism and is formed through the oxidation of suberyl-CoA, an intermediate of fatty acid metabolism. Suberylglycine is primarily associated with a group of inherited metabolic disorders known as organic acidemias, leading to the accumulation of various organic acids, including suberylglycine, in the body. The measurement of suberylglycine levels in biological samples, such as urine or blood, is used as a diagnostic marker for certain organic acidemias (Divry et al., 1984; Rinaldo et al., 1989). The elevated urinary levels of suberylglycine have been associated with hereditary medium-chain acyl-CoA hydrogenase (MCAD) failing (Rinaldo et al., 1989); however, in the current case, metabolite levels were found to be decreased in the liver. MCAD is suggested to be the most common cause of nonalcoholic fatty liver disease (Prieto Jimenez et al., 2022). Prieto Jimenez et al. (2022) have reported rare hepatic steatosis secondary to the chronic case, expressed in an infant, where the levels of circulatory suberylglycine were elevated. This early observation of potential CSM-induced MCAD deficiency is key evidence and should be further investigated.

### 4.5 Downregulation of liver spermine

Spermine was also found to be downregulated in the liver by the increase in CMS. The compound is a polyamine, naturally present in cells and tissues of living organisms, including humans. It is derived from the amino acid ornithine through a series of enzymatic reactions. Spermine plays important roles in various biological processes, including cell growth, proliferation, and DNA stabilization (Zhou et al., 2018). It has been found that in the plasma of patients with chronic renal failure, the circulatory levels of spermine are decreased (Igarashi et al., 2006). In our study, the hepatic levels of spermine were decreased by the increased CMS dose, but there were no detected alterations of this metabolite in plasma. Interestingly, polyamines such as spermine are proved to counterbalance drug adverse effects, such as hepatotoxicity, and thus are administrated as protective agents (Zhou et al., 2018), inhibiting cell apoptosis (Amin et al., 2021). In the current case, the CMS-induced decrease of spermine indicates that the drug prohibits homeostatic mechanisms, such as those that counterbalance the drug’s effects.

### 4.6 Disturbed purine metabolism and renal dysfunction

**FIGURE 7:**
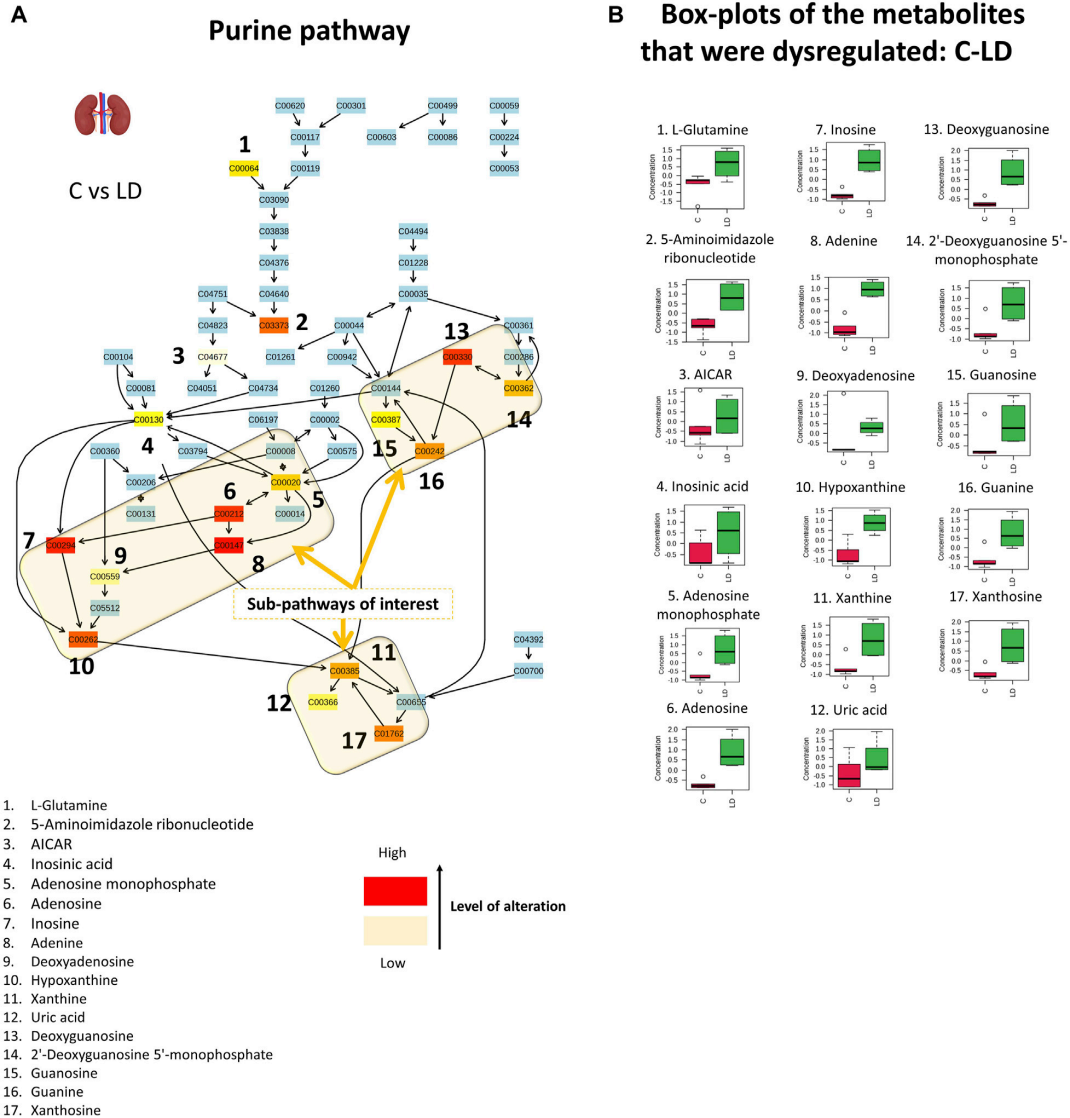
Graphical description of the alteration that occurred in renal purine metabolism with the administration of 1.0-mg/kg CMS. The sub-pathways of interest point out the locations where sequential alterations were observed. The box-plots show the normalized content of the perturbated metabolites in the C (red) and LD (green) groups.

The results of the semi-quantitive pathway analysis of the C–LD group are described in Figure 7 where, the altered metabolites of the pathway are shown in Figure 7A and the box-plots of their corrected signal are shown in Figure 7B. From the detected metabolites, 17 were increased by the low CMS dose, with the main perturbations occurring in the sub-pathways that result in the formation of xanthine, uric acid (UA), and guanine (Figure 7). The dysregulation of nine sequential metabolites, in the sub-pathway of UA formation, provides strong proof that CMS causes severe effects on renal purine metabolism. An intriguing observation is that in the C–LD comparison, the purine metabolism pathway displayed critical alterations [*p* (FDR) = 0.04], whereas in the LD–HD comparison, there were no important differences detected in metabolite levels [*p* (FDR) = 0.37, Supplementary Figure S4].

Purines are enzymatically transformed into hypoxanthine and then into xanthine, which is the precursor of UA (Figure 7). The formation of UA is followed by the generation of superoxide anions and reactive oxygen species, as degradation byproducts. The extended production of UA, as observed in the current case, leads to high levels of intracellular oxidative stress-inducing factors causing cell damage (Gherghina et al., 2022). Dysregulation of purine metabolism, particularly hyperuricemia (elevation of UA), is associated with kidney injury and is a marker for the progression of CKD (Jiao et al., 2022). There are several proposed associations between UA and kidney impairment: 1) formation of monosodium urate crystals that precipitate in the tubules of the extrarenal system; 2) oxidative stress due to the intracellular pro-oxidative properties of UA that cause endothelial dysfunction, renal fibrosis, inflammation, and glomerulosclerosis; and 3) UA prevents nitric oxide (NO) synthesis and thus hinders endothelial cell proliferation (Gherghina et al., 2022; Jiao et al., 2022). The increased levels of xanthine and UA are also associated with aging-induced renal impairment (Jiao et al., 2022).

Additionally, the alterations of purine metabolism could be the key point regarding the connection of neuro- and nephrotoxicity occurring due to CMS administration. It is proposed that decreased eGFR leads to an increase in circulatory levels of metabolic waste (uremic toxins and kidney hormones), provoking all types of neurological complications by triggering the nervous system (dopaminergic system) and causing brain dysfunction (Mazumder et al., 2018; De Donato et al., 2022). In addition, there are several reports of cognitive impairment in patients with CKD (Mazumder et al., 2018). A recent computational docking study tested the binding affinity of xanthine, hypoxanthine, UA, and 2,8-DHA with acetylcholinesterase (AChE): UA showed a higher binding affinity, and xanthine and hypoxanthine presented high docking scores as well (Mazumder et al., 2018). AChE hydrolyzes acetylcholine, which is important for learning and memory, so its rapid degradation by AChE leads to dementia and Cognitive Impairment (CI) (Wamser et al., 2013). *In vitro* testing showed that hypoxanthine enhanced the action of AChE (Wamser et al., 2013). This observation indicates a naïve correlation between CMS-neurotoxicity and CMS-nephrotoxicity that should be further investigated.

The results of the current study are in accordance with the results of Nguyen et al.’s corresponding metabolomics study, in which ∼16-fold higher doses of CMS were administered, i.e., 25 mg/kg and 50 mg/kg (Long et al., 2022) as low and high dose, respectively, *in vivo*. Nguyen et al. observed critical separation trends (PCA) between the control and the 25-mg/kg/day-treated mice and dose-responding alterations in the metabolomics profiles of kidney and liver tissues. Moreover, comparing the kidneys and livers of the control and treated mice, they observed differences in pathways related to antioxidation mechanisms, i.e., glutathione metabolism, and in pathways related to DNA synthesis such as purine metabolism. In addition, the study indicated CMS-induced oxidative stress and caspase-dependent cell apoptosis at the renal level.

In the current study, important separation trends were observed even in the kidneys of the LD group (1.0 mg/kg/day) versus the control, indicating that CMS has a critical impact on renal tissue, even at these doses. Therefore, the determination of early biomarkers will help in the detection of latent kidney dysfunction, even before it is expressed with clinical symptoms, and it will permit the timely adjustment of the curation protocol too. The dose–response metabolomics alterations are also confirmed for the lower dose ranges, as shown by the PLS regression results of the current study. Moreover, there are early indications of increased oxidative stress in kidney and liver tissue, i.e., the upregulation of M*N*RSal renal levels that induces cell apoptosis by activating caspase-3. Finally, the current study showed purine metabolism alterations induced by low CMS that were not further amplified by the 0.5-mg/kg/day increase in the dose. However, the Nguyen et al. study showed that the purine metabolism is related to a higher dose increase, and therefore, further investigation of this pathway’s alterations at a wider range of doses, i.e., 1–5 mg/kg/day would provide solid information regarding CMS impact on DNA synthesis and would also shed light on naïve assumption of nephro- and neurotoxicity.

## 5 Conclusion

CMS is the last-resort antibiotic factor administered for the treatment of infections caused by multidrug-resistant bacteria. However, the use of the drug is attenuated by the occurrence of neurological and renal complications resulting from its administration. Several studies have focused on CMS-induced neuro- and nephrotoxicity by administrating high doses of the drug to simulate the toxic condition. The current study aimed to shed light on the biochemical alterations triggered by the recommended human dose, 1–1.5 mg/kg/day. Moreover, the study investigated the impact of the drug on the circulatory system, and on the renal and liver function as well. So far, despite the existence of indications regarding CMS-induced hepatotoxicity, there is no evidence for the displaying mechanisms. The study showed that even the lower human dose (1 mg/kg) had a severe impact on the kidneys and also pointed out a linear response between the drug dose and the metabolic alterations for the plasma, kidney, and liver. Sixteen variables showed significant correlation to the dose, and six of them were identified: suberylglycine (liver, ↓), spermine (liver, ↓), (R)-N-methylsalsolinol (kidney, ↑), phenylacetic acid (kidney, ↓), 2,8-dihydroxyadenine (kidney, ↑), and dopamine-4-sulfate (kidney, ↑). In summary, the results of the current study showed that CMS

• induces the renal dopamine pathway;
• increases the renal levels of 2,8-DHA and probably leads to the formation and precipitation of urinary crystals and kidney stones;
• disrupts the renal purine metabolism, increasing the formation of xanthine, hypoxanthine, and UA. Xanthine is considered an AChE activator, leading to the rapid degradation of acetylcholine. This is strong evidence for the shared metabolic background of CMS-induced nephrotoxicity and neurotoxicity;
• disrupts hepatic MCAD, probably leading to hepatic steatosis; and
• decreases hepatic levels of spermine, which counterbalances hepatotoxicity by inhibiting cell death.

Overall, the current study, investigating the impact of CMS doses that correspond to those applied in clinical practice, revealed that these doses trigger biochemical pathways related to kidney and liver toxicity. These alterations are early detectable, even when there is no clinical evidence of toxicity, and could facilitate the adjustment of the curation scheme in the future.

### 5.1 Limitations to the study

The current study is impacted by potential limitations; i.e., DIA MS acquisition, despite being a more informative methodology, restricted the ability to perform more extended identification. Moreover, the study focused on nephrotoxicity and hepatotoxicity caused by CMS and did not examine other organs such as the brain and the heart. In particular, the examination of metabolomics alteration in the brain could amplify the assumption of a correlation between nephrotoxicity and neurotoxicity and will be an objective of a future study. The study did not consider other toxicological endpoints, i.e., histological signs, and therefore, the metabolomics evidence is not correlated with the clinical picture of the mice. Finally, despite that the metabolites are molecules of universal structure, the exact extrapolation of the effects from humans to animals might not be accurate enough to draw a safe conclusion and extend it to the mechanism proposed. Nevertheless, the indications of this study set the basis for a thorough investigation of CMS toxicity.

## Data Availability

The datasets presented in this study can be found in online repositories. The names of the repository/repositories and accession number(s) can be found at: https://www.ebi.ac.uk/metabolights/, MTBLS8898.
