# Supplementary material for: Metabolomics highlights biochemical perturbations occurring in the kidney and liver of mice administered a human dose of colistin
Source: Front Mol Biosci. 2024 Jul 10;11:1338497. doi: 10.3389/fmolb.2024.1338497 (PMC11266156; doi:10.3389/fmolb.2024.1338497)
Supplement: Supplementary file 1 [file DataSheet1.docx]

Supplementary Material

# Supplementary Data

## RPLC Chromatography Conditions

#### Mobile phases for ESI (+):

A: 0.01% Formic Acid- 5mM Ammonium Formate 90:10 Water: Methanol,

B: 0.01% Formic Acid- 5mM Ammonium Formate Methanol

#### Mobile phases for ESI (-):

A: 5mM Ammonium Formate 90:10 Water: Methanol,

B: 5mM Ammonium Methanol

#### Gradient for ESI(+) & ESI(-):

0-1 min: 1% B,

12 min: 100% B

12.1 min: 1% B

12.2-15: 1% B

Column Temperature: 30 ^o^C

## Data pre-processing

The QCRFSC algorithm was employed for QC based signal-correction. The NA.filter was set at 0.8 in order to include only the variables with non-zero values for the 80% of the samples. The %CV cutoff was set at 40 to exclude the variables with relative standard deviation (RSD) higher than 40%. Afterwards, yohimbine and reserpine were used for IS-based signal correction, employing the normalization using optimal selection of multiple internal standards (NOMIS) algorithm playing in NOREVA2.0 platform. Then, the data were transformed (LogTranformation), and scaled (Unite Variance scaling) in order to ensure their normality.

# Supplementary Figures and Tables

## Supplementary Figures


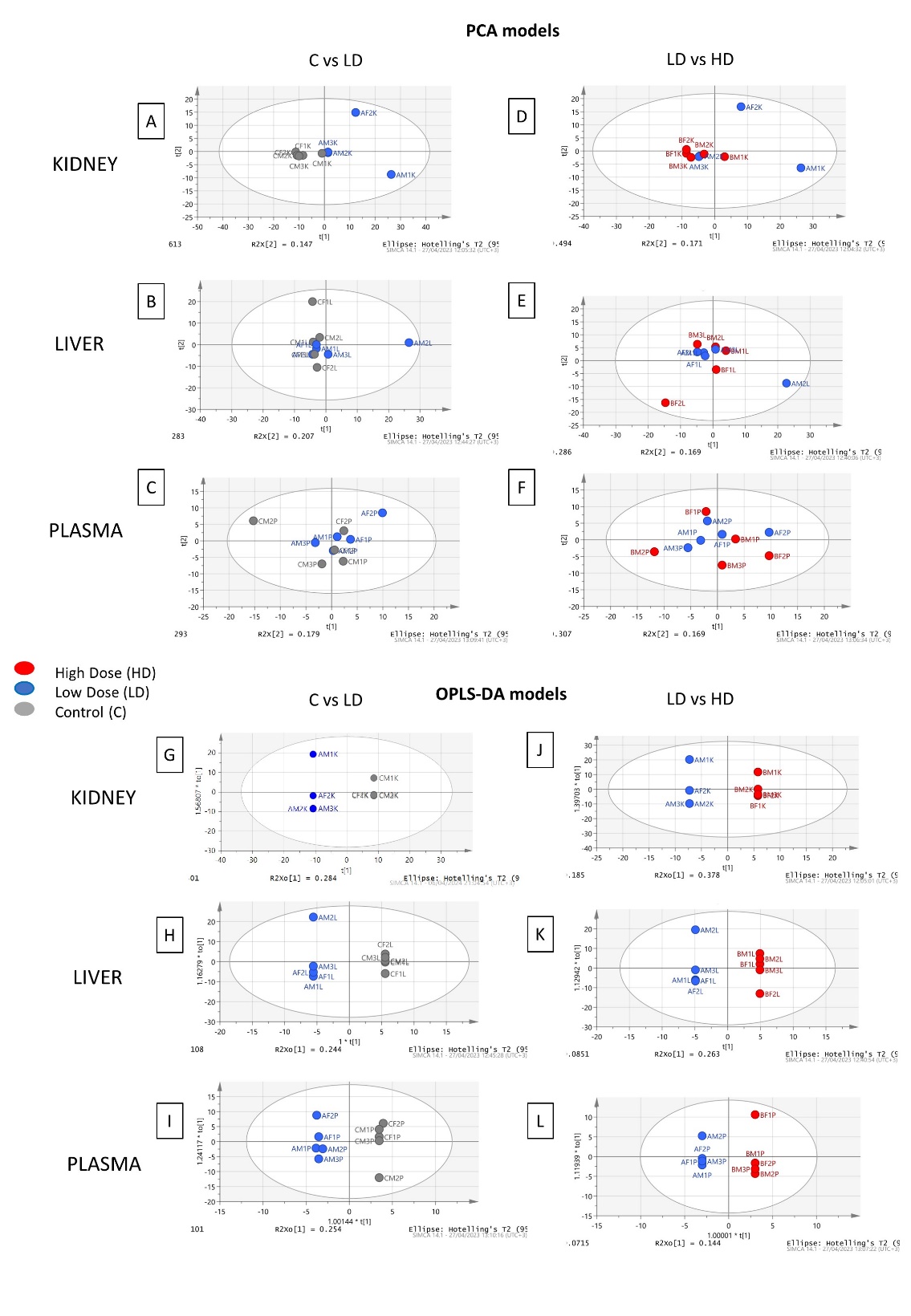


**Supplementary Figure 1.** Summary of the PCA and OPLS-DA results of the ESI- datasets. PCA scores’ plots of: (A) Kidney, C (n=5)-LD (n=5); (B) Liver, C (n=5)-LD (n=5); (C) Plasma, C (n=5)-LD (n=5); (D) Kidney, LD (n=5)-HD (n=5); (E) Liver, LD (n=5)-HD (n=5); (F) Plasma, LD (n=5)-HD (n=5). OPLS-DA scores’ plots: (G) Kidney, C (n=5)-LD (n=4); (H) Liver, C (n=5)-LD (n=5); (I) Plasma, C (n=5)-LD (n=5); (J) Kidney, LD (n=5)-HD (n=5); (K) Liver, LD (n=5)-HD (n=5); (L) Plasma, LD (n=5)-HD (n=5).


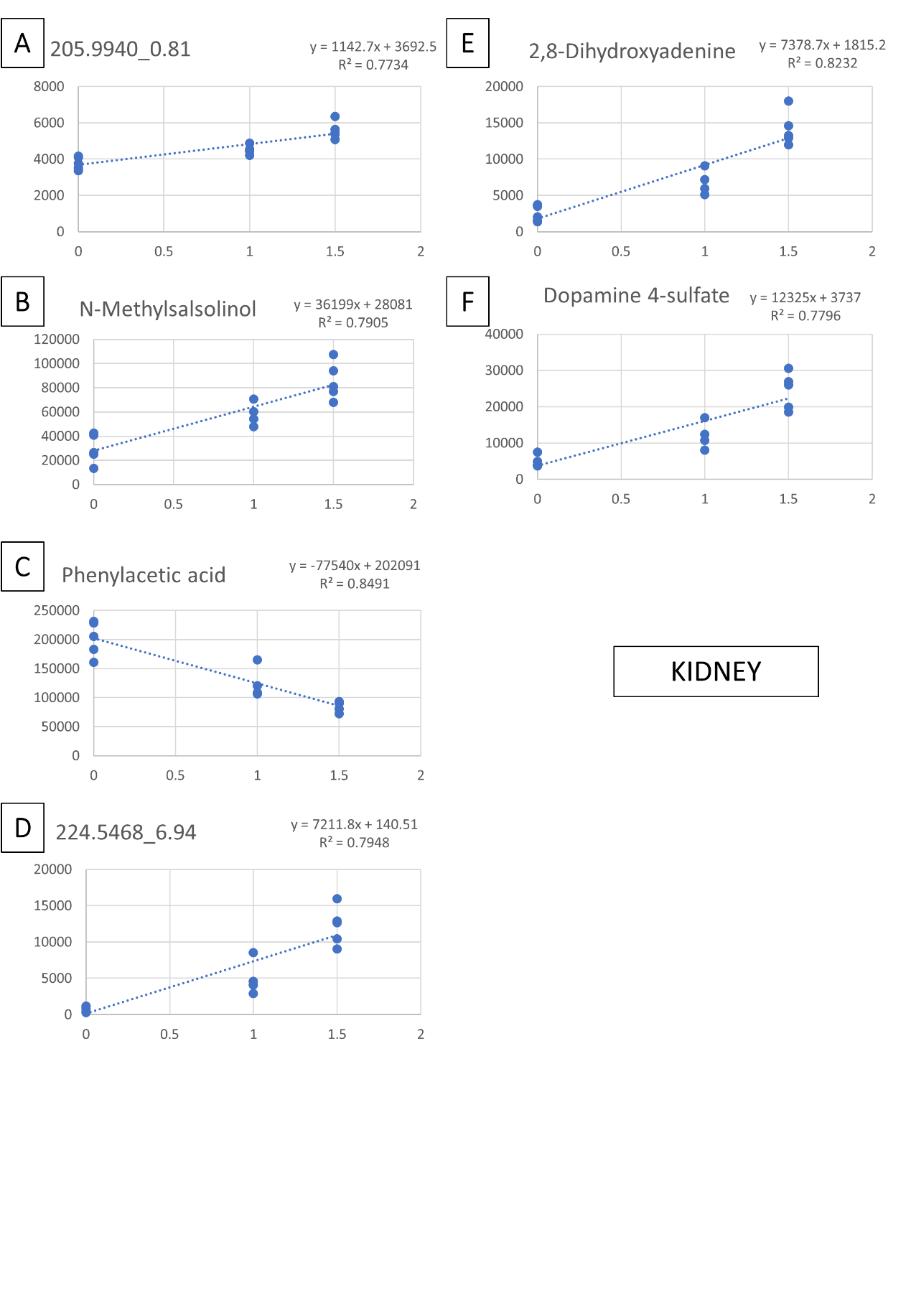
**Supplementary Figure 2.** Regression curves of the dose-response variables, resulted by the SUS-plot procedure for the kidney dataset.


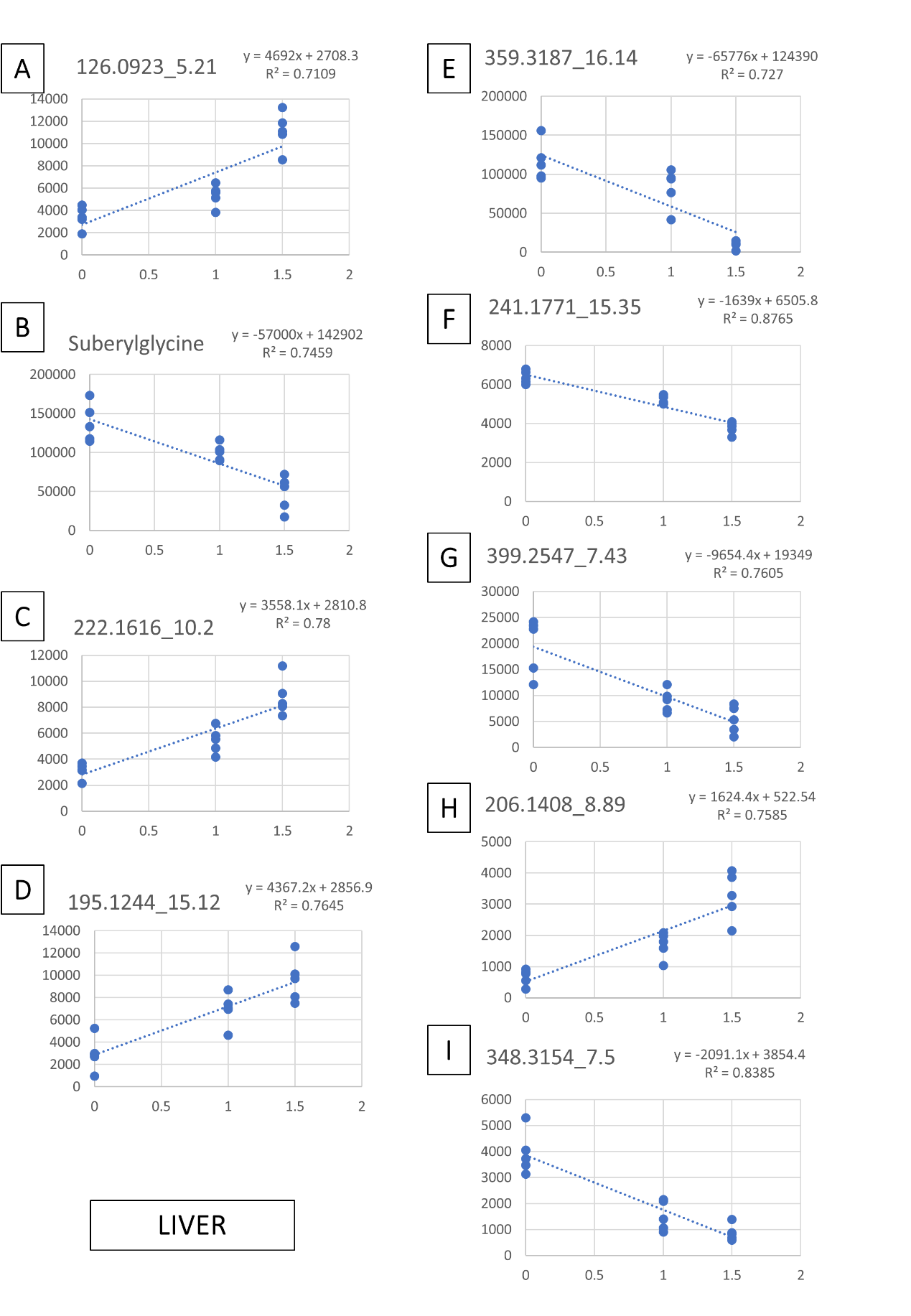


**Supplementary Figure 3.** Regression curves of the dose-response variables, resulted by the SUS-plot procedure for the liver dataset.


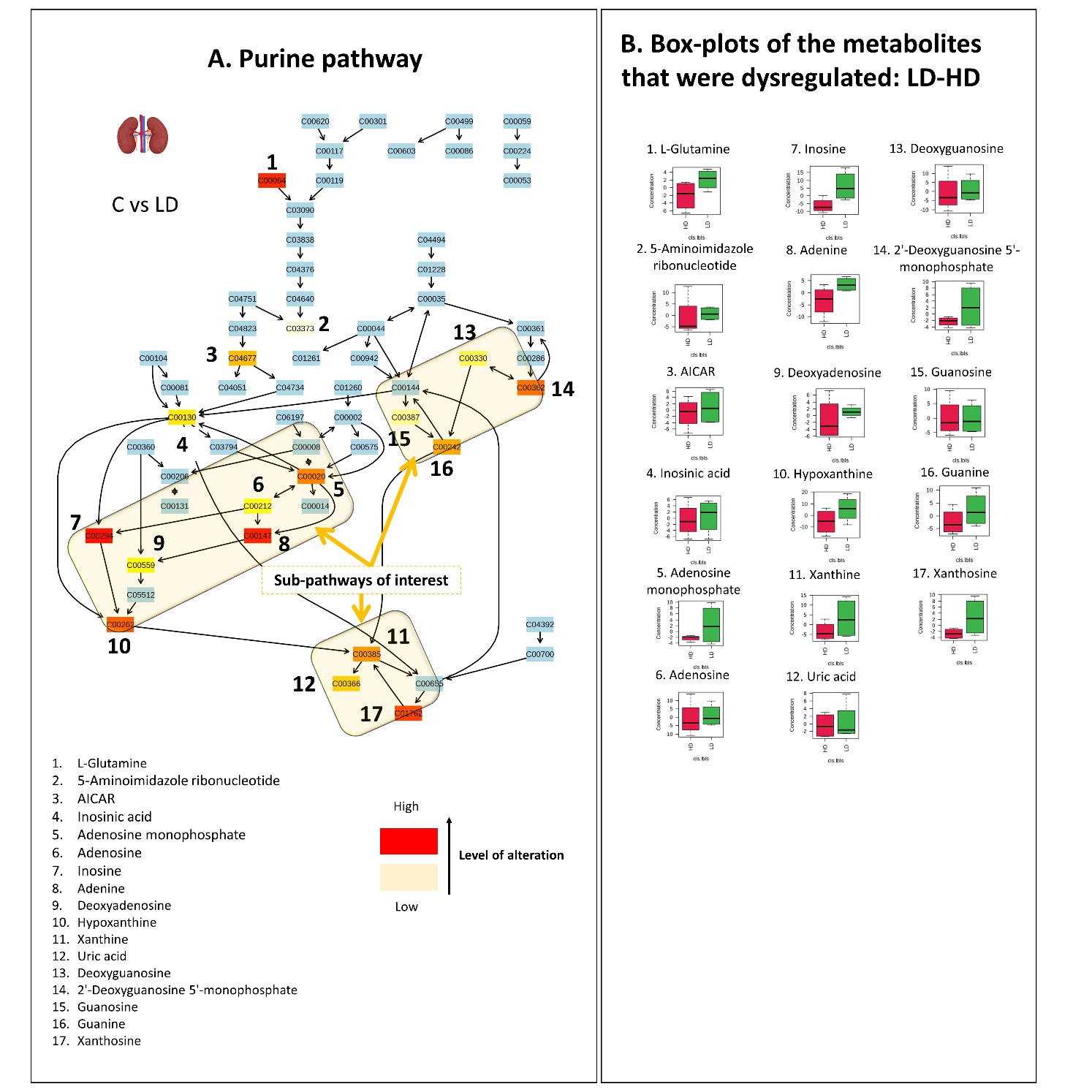


**Supplementary Figure 4.** Graphical description of the alteration occurred in renal purine metabolism with the increase of CMS dose. The sub-pathways of interest point out the locations that sequential alteration were observed. The boxplots show the normalized content of the perturbated metabolites in HD (red) and LD (green) groups.

## Supplementary Tables

**Supplementary Table 1.** Set up conditions of electrospray ionization source (ESI) and for the quadruple- time-of-flight mass spectrometer (QTOFMS)

|  | **RPLC (+)** | **RPLC (-)** |
| --- | --- | --- |
| **Capillary Voltage** | 2500 V | 3000 V |
| **End plate offset** | 500 V | 500 V |
| **Nebulizer (N_2_)** | 2 bar | 2 bar |
| **Drying gas (N_2_)** | 10 L/min | 10 L/min |
| **Drying temp.** | 200 ^o^C | 200 ^o^C |
| **Low CE** | 4 V | 4 V |
| **High CE** | 4-25 V | 4-25 V |

**Supplementary Table 2.** Summary of the figures of merit and of the prediction ability of the generated models

| SAMPLE | ESI | COMPARISON | R2y | Q2 | R2X-perm | Q2-perm | MODELS ROC | MISSCLASSIFICATION ERROR (%) |
| --- | --- | --- | --- | --- | --- | --- | --- | --- |
| KIDNEY | + | C-LD | 1 | 1 | 1 | 0.99 | 1 | 0 |
|  |  | LD-HD | 1 | 1 | 1 | 0.22 | 1 | 0 |
|  | - | C-LD | 1 | 1 | 1 | 0.74 | 1 | 0 |
|  |  | LD-HD | 1 | 1 | 0.96 | 1 | 1 | 0 |
| LIVER | + | C-LD | 1 | 1 | 1 | 0.99 | 1 | 0 |
|  |  | LD-HD | 1 | 1 | 1 | 0.99 | 1 | 0 |
|  | - | C-LD | 1 | 1 | 1 | 0.99 | 1 | 0 |
|  |  | LD-HD | 1 | 1 | 1 | 0.92 | 1 | 0 |
| PLASMA | + | C-LD | 1 | 1 | 1 | 0.97 | 1 | 0 |
|  |  | LD-HD | 1 | 1 | 1 | 1 | 1 | 0 |
|  | - | C-LD | 1 | 1 | 1 | 0.97 | 1 | 0 |
|  |  | LD-HD | 1 | 1 | 1 | 1 | 1 | 0 |

**Supplementary Table 3.** The list of m/z of metabolites that participate in purine’s metabolism and were used for targeted peak-picking in the raw data of the kidney ESI+ dataset.

| **m/z** | **name** | **formula** | **Neutral formula** |
| --- | --- | --- | --- |
| 136.061772 | Adenine | C5H6N5^1+ | C5H5N5 |
| 560.078951 | Adenosine diphosphate ribose | C15H24N5O14P2^1+ | C15H23N5O14P2 |
| 18.033826 | Ammonia | NH4^1+ | NH3 |
| 44.997106 | Carbon dioxide | CHO2^1+ | CO2 |
| 152.056686 | Guanine | C5H6N5O^1+ | C5H5N5O |
| 35.012756 | Hydrogen peroxide | H3O2^1+ | H2O2 |
| 137.045787 | Hypoxanthine | C5H5N4O^1+ | C5H4N4O |
| 19.017841 | Water | H3O^1+ | H2O |
| 32.997106 | Oxygen | HO2^1+ | O₂ |
| 98.984172 | Phosphoric acid | H4O4P^1+ | H3PO4 |
| 169.035616 | Uric acid | C5H5N4O3^1+ | C5H4N4O3 |
| 153.040702 | Xanthine | C5H5N4O2^1+ | C5H4N4O2 |
| 745.090555 | NADP | C21H30N7O17P3^1+ | C21H29N7O17P3 |
| 664.116399 | NAD | C21H28N7O17P3^1+ | C21H27N7O14P2 |
| 508.003023 | Adenosine triphosphate | C10H17N5O13P3^1+ | C10H16N5O13P3 |
| 147.076419 | L-Glutamine | C5H11N2O3^1+ | C5H10N2O3 |
| 269.088046 | Inosine | C10H13N4O5^1+ | C10H12N4O5 |
| 428.036692 | Adenosine diphosphate | C10H16N5O10P2^1+ | C10H15N5O10P2 |
| 330.059797 | cAMP | C10H13N5O6P^1+ | C10H12N5O6P |
| 348.070361 | Adenosine monophosphate | C10H15N5O7P^1+ | C10H14N5O7P |
| 284.098945 | Guanosine | C10H14N5O5^1+ | C10H13N5O5 |
| 364.065276 | Guanosine monophosphate | C10H15N5O8P^1+ | C10H14N5O8P |
| 523.997938 | Guanosine triphosphate | C10H17N5O14P3^1+ | C10H16N5O14P3 |
| 390.959093 | Phosphoribosyl pyrophosphate | C5H14O14P3^1+ | C5H13O14P3 |
| 349.054377 | Inosinic acid | C10H14N4O8P^1+ | C10H13N4O8P |
| 508.987039 | Inosine triphosphate | C10H16N4O14P3^1+ | C10H15N4O14P3 |
| 444.031607 | Guanosine diphosphate | C10H16N5O11P2^1+ | C10H15N5O11P2 |
| 127.061437 | 5-Aminoimidazole-4-carboxamide | C4H7N4O^1+ | C4H6N4O |
| 332.075447 | Deoxyadenosine monophosphate | C10H15N5O6P^1+ | C10H14N5O6P |
| 252.109116 | Deoxyadenosine | C10H14N5O3^1+ | C10H13N5O3 |
| 492.008108 | Deoxyadenosine triphosphate | C10H17N5O12P3^1+ | C10H16N5O12P3 |
| 148.060434 | L-Glutamic acid | C5H10NO4^1+ | C5H9NO4 |
| 268.10403 | Adenosine | C10H14N5O4^1+ | C10H13N5O4 |
| 285.082961 | Xanthosine | C10H13N4O6^1+ | C10H12N4O6 |
| 253.093131 | Deoxyinosine | C10H13N4O4^1+ | C10H12N4O4 |
| 348.070361 | 2'-Deoxyguanosine 5'-monophosphate | C10H15N5O7P^1+ | C10H14N5O7P |
| 508.003023 | dGTP | C10H17N5O13P3^1+ | C10H16N5O13P3 |
| 339.070027 | AICAR | C9H16N4O8P^1+ | C9H15N4O8P |
| 365.049291 | Xanthylic acid | C10H14N4O9P^1+ | C10H13N4O9P |
| 346.054711 | Guanosine 2',3'-cyclic phosphate | C10H13N5O7P^1+ | C10H12N5O7P |
| 474.173173 | 10-Formyltetrahydrofolate | C20H24N7O7^1+ | C20H23N7O7 |
| 315.058794 | 5'-Phosphoribosyl-N-formylglycinamide | C8H16N2O9P^1+ | C8H15N2O9P |
| 455.080986 | SAICAR | C13H20N4O12P^1+ | C13H19N4O12P |
| 285.048229 | Glycineamideribotide | C7H14N2O8P^1+ | C7H13N2O8P |
| 296.064213 | 5-Aminoimidazole ribonucleotide | C8H15N3O7P^1+ | C8H14N3O7P |
| 869.045372 | Diguanosine tetraphosphate | C20H29N10O21P4^1+ | C20H28N10O21P4 |
| 367.064942 | Phosphoribosyl formamidocarboxamide (FAICAR) | C10H16N4O9P^1+ | C10H15N4O9P |
| 268.10403 | Deoxyguanosine | C10H14N5O4^1+ | C10H13N5O4 |
| 664.116399 | NADH | C21H28N7O14P2^1+ | C21H27N7O14P2 |
| 231.026431 | D-Ribose 5-phosphate | C5H12O8P^1+ | C5H11O8P |
| 428.036692 | dGDP | C10H16N5O10P2^1+ | C10H15N5O10P2 |
| 231.026431 | Ribose 1-phosphate | C5H12O8P^1+ | C5H11O8P |
| 215.031516 | Deoxyribose 1-phosphate | C5H12O7P^1+ | C5H11O7P |
| 230.042415 | 5-Phosphoribosylamine | C5H13NO7P^1+ | C5H12NO7P |
| 464.08132 | Adenylosuccinic acid | C14H19N5O11P^1+ | C14H18N5O11P |
| 314.074778 | Phosphoribosylformylglycinamidine | C8H17N3O8P^1+ | C8H16N3O8P |
| 429.020708 | IDP | C10H15N4O11P2^1+ | C10H14N4O11P2 |
| 76.039305 | Glycine | C2H6NO2^1+ | C2H5NO2 |
| 446.178258 | Tetrahydrofolic acid | C19H24N7O6^1+ | C19H23N7O6 |
| 117.018235 | Fumaric acid | C4H5O4^1+ | C4H4O4 |
| 745.090555 | NADPH | C21H30N7O17P3^1+ | C21H29N7O17P3 |
| 134.044784 | L-Aspartic acid | C4H8NO4^1+ | C4H7NO4 |
| 412.041778 | dADP | C10H16N5O9P2^1+ | C10H15N5O9P2 |
| 121.064791 | Phenylacetaldehyde | C8H9O^1+ | C8H8O |
| 166.086255 | L-Phenylalanine | C9H12NO2^1+ | C9H11NO2 |
| 122.096426 | Phenethylamine | C8H12N^1+ | C8H11N |
| 164.046796 | Phenylpyruvate | C9H8O3^1+ | C9H7O3 |
| 872.148701 | Benzoyl-CoA | C28H41N7O17P3S^1+ | C28H40N7O17P3S |
| 137.059706 | Phenylacetic acid | C8H9O2^1+ | C8H8O2 |
| 153.054621 | 2-Hydroxyphenylacetate | C8H9O3^1+ | C8H8O3 |
| 165.054621 | 2-Hydroxy-3-phenylpropenoate | C9H9O3^1+ | C9H8O3 |
| 180.06552 | Hippurate | C9H10NO3^1+ | C9H9NO3 |
| 182.08117 | L-Tyrosine | C9H12NO3^1+ | C9H11NO3 |
